# *Helicobacter pylori* Eradication Prevents Metachronous Gastric Neoplasms after Endoscopic Resection of Gastric Dysplasia

**DOI:** 10.1371/journal.pone.0143257

**Published:** 2015-11-18

**Authors:** Seung Hwan Shin, Da Hyun Jung, Jie-Hyun Kim, Hyun Soo Chung, Jun Chul Park, Sung Kwan Shin, Sang Kil Lee, Yong Chan Lee

**Affiliations:** 1 Department of Internal Medicine, Yonsei University College of Medicine, Seoul, Korea; 2 Department of Internal Medicine, Gangnam Severance Hospital, Yonsei University College of Medicine, Seoul, Korea; National Cancer Center, JAPAN

## Abstract

**Purpose:**

There is insufficient data about the role of eradication of *H*. *pylori* after endoscopic resection (ER) for gastric dysplasia. The aim was to investigate the benefit of *H*. *pylori* eradication after ER in patients with gastric dysplasia to prevent metachronous gastric neoplasms.

**Materials and Methods:**

We retrospectively reviewed 1872 patients who underwent ER of gastric dysplasia. We excluded patients with a follow-up period of <2 years or who had not undergone tests for active *H*. *pylori* infection. A total of 282 patients were enrolled. The patients were categorized into those without active *H*. *pylori* infection (*H*. *pylori*-negative group, n = 124), those who successfully underwent *H*. *pylori* eradication (eradicated group, n = 122), and those who failed or did not undergo *H*. *pylori* eradication (persistent group, n = 36).

**Results:**

Metachronous recurrence was diagnosed in 36 patients, including 19 in the *H*. *pylori*-negative group, 10 in the eradicated group, and 7 in the persistent group. The cumulative incidence of metachronous recurrence was significantly lower in the *H*. *pylori*-eradicated group in comparison with either of the *H*. *pylori*-persistent (non-eradicated or failed) groups (*p* = 0.039). Similarly, the incidence of metachronous recurrence was significantly lower in the *H*. *pylori*-eradicated group compared with the *H*. *pylori*-negative group (*p* = 0.041).

**Conclusion:**

Successful *H*. *pylori* eradication may reduce the development of metachronous gastric neoplasms after ER in patients with gastric dysplasia.

## Introduction

*Helicobacter pylori* (*H*. *pylori*) infection is a group I carcinogen for gastric cancer as defined by the International Agency for Research on Cancer (IARC), a subdivision of the World Health Organization (WHO) [1]. Correa *et al*. contend that *H*. *pylori* infection is closely associated with progression to gastric atrophy, intestinal metaplasia (IM), dysplasia, and cancer [2]. The reported progression rate of atrophic gastritis, intestinal metaplasia and dysplasia to gastric cancer varies from 0 to 1.8%, 0 to 10%, and 0 to 73% per year, respectively [3]. Male gender is the significant risk factor for the development of gastric cancer, with a nearly 2:1 male to female dominance [4]. Previous studies showed that seroprevalence of *H*. *pylori* was higher in males than in females [5–7]. Currently, endoscopic resection (ER) is the accepted standard treatment for selected cases of early gastric cancer (EGC) in Korea. Because gastric dysplasia is a precancerous lesion, this pathology also can be a candidate for ER, which has the additional benefit of providing a histologic diagnosis that could result in an upgrade from biopsy-proven dysplasia to cancer [8]. In Korea, ER is widely used for the treatment of gastric dysplasia and EGC [9]. ER can preserve the stomach; however, metachronous gastric cancer may develop in the stomach remnant after the procedure [10,11]. For this reason, endoscopic surveillance for metachronous gastric neoplasms has become an emerging issue during the follow-up interval after ER. Some studies have shown that the eradication of *H*. *pylori* after ER is helpful to prevent the development of metachronous gastric cancer [12–14]; however, several other studies have failed to show a prophylactic benefit for *H*. *pylori* eradication [15,16]. At present, the guidelines for the treatment *H*. *pylori* infection in Korea recommends eradication of *H*. *pylori* after ER of EGC according to the findings of positive studies [17]. However, there is insufficient data about the role of eradication of *H*. *pylori* after ER for the treatment of gastric dysplasia. The aim of this study was to determine whether *H*. *pylori* eradication prevents the development of metachronous gastric neoplasms in patients with gastric dysplasia following ER.

## Methods

### Patients

**Fig 1:**
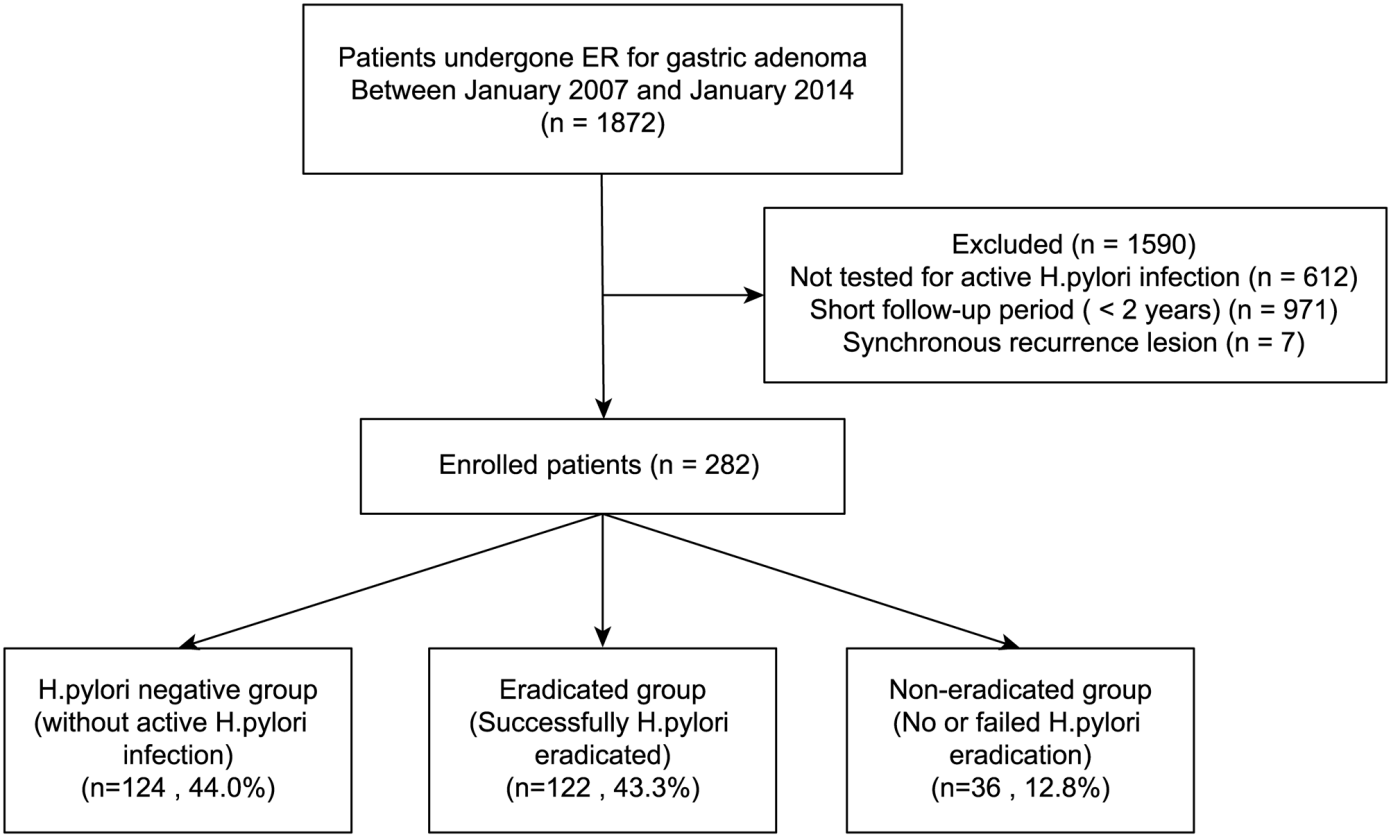
A flow chart of patient enrollment.

Between January 2007 and January 2014, 1872 patients were diagnosed with low- and high-grade gastric dysplasia and underwent ER at Severance Hospital, Seoul, Korea. Of these patients, we excluded 612 patients who did not undergo tests for active *H*. *pylori* infection including a urea breath test, a rapid urease test, or histopathological examination. We also excluded 971 patients with only a short-term follow-up period (<2 years) and 7 patients who had a recurrence in a previous ER site. Ultimately we enrolled a total of 282 patients for analysis in this study. The ER procedures were performed by expert endoscopists who had a previous experience of over 1000 gastric endoscopy cases per year. The patients were divided into three groups according to presence of active *H*. *pylori* infection and successful eradication. The patient selection and grouping flow diagram is shown in Fig 1: (1) those without active *H*. *pylori* infection at the time of ER (the *H*. *pylori*-negative group, n = 124), (2) those with a successfully treated *H*. *pylori* infection (the *H*. *pylori*-eradicated group, n = 122), (3) those who failed treatment of *H*. *pylori* infection or were untreated (the *H*. *pylori-*persistent group, n = 36). A time interval of at least 6 months was observed between *H*. *pylori* treatment and the development of metachronous gastric neoplasms to accurately assess the effect of *H*. *pylori* eradication on the incidence of metachronous gastric lesions after ER. Patients were evaluated in terms of their clinicopathological characteristics including age, gender, tumor location, gross appearance, histological type, tumor size, multiplicity of tumors, and the presence of co-morbid ulcers such as gastric ulcer or duodenal ulcer. The definition of smoking and alcohol drinking history were divided into yes or no. The clinicopathological characteristics were analyzed retrospectively from patient medical records. The Institutional Review Board (IRB) of Severance Hospital approved this study. We received a consent exemption from the IRB. Patients records and information was anonymized.

### Evaluation of *H*. *pylori* infection status and treatment

To determine the *H*. *pylori* infection status rapid urease and urea breath tests and histological assessment were used. Patients who showed negative results for all three examinations were assigned to the *H*. *pylori*-negative group. The patients who had a positive result for at least one test among these were assigned to the active *H*. *pylori* infection group. The active *H*. *pylori* infection group was subdivided into two groups depending on their infection treatment outcome and assigned to either the eradicated or persistent groups. *H*. *pylori* eradication therapy was carried out in accordance with the guideline for treatment of *H*. *pylori* infection in Korea [17]. Of the total 282 patients, 122 patients assigned to the eradicated group received triple therapy of a standard dose of a proton pump inhibitor (PPI), 500 mg of clarithromycin, and 1000 mg of amoxicillin twice a day for 7 days. Twelve patients in the eradicated group who initially failed eradication after conventional triple therapy underwent a secondary regimen of bismuth-based quadruple therapy.

### Follow-up schedule after the endoscopic resection

Follow-up endoscopic examinations with endoscopy and biopsy were conducted at regular intervals after endoscopic resection (3 and 12 months and annually after the first year of follow-up). A biopsy was performed at the previous ER site and any other sites suspected to represent a recurrence of metachronous neoplasm.

### Histopathological evaluation of the gastric lesion

Biopsy or resection specimens were examined by two expert pathologists. The histology of gastric cancers were reviewed according to the new Japanese classification for gastric carcinomas [18] and the grade of gastric dysplasia was categorized according to the Vienna classification [19]. A metachronous neoplasm was defined as evident dysplasia or carcinoma that developed subsequently (i.e., more than 6 months after ER of the primary gastric dysplasia).

### Statistical analysis

The chi-square test and Fisher’s exact test were used to compare the clinicopathological factors between the groups according to the presence/absence of active *H*. *pylori* infection and failed/successful eradication. The Student’s *t*-test was used for non-categorical variables in the intergroup comparisons of clinicopathological characteristics. The threshold for statistical significance was set at *p* < 0.05. The incidence of metachronous gastric neoplasms was calculated by the Kaplan-Meier method, and compared among the three groups by the log-rank test. A Cox proportional hazards model and multivariate analyses were used for risk assessment. Statistical analyses were performed using the Statistical Package for Social Sciences Version 18.0 (SPSS Inc., Chicago, IL, USA).

## Results

### Baseline characteristics of the study population

The baseline characteristics of the three groups are summarized in Table 1. The enrolled patients included 200 (70.9%) males and 82 (29.1%) females and the median age was 68 years old. There were no significant differences among the three groups in the sex ratio, smoking and alcohol history, previous ulcer history and histology, or the location and size of the tumor. The clinicopathological characteristics of patients according to the development of metachronous neoplasms are shown in Table 2. Among the 282 patients, metachronous recurrence developed in 36 (14.6%) patients after ER of gastric dysplasia. There were no significant differences between the two groups in the sex ratio, smoking and alcohol consumption history, initial histology, lesion size, or location of the tumor. The metachronous group was significantly older than the non-metachronous group (*p* = 0.035).

**Table 1:** Baseline characteristics of enrolled patients.

Characteristic	*H*. *pylori* negative group (n = 124, %)	Eradicated group (n = 122, %)	Persistent group (n = 36, %)	*P*-value
Mean age (year) ^a^	68.89 ± 8.78	66.64 ± 8.55	67.61 ± 8.49	0.126
Sex				0.621
Male	91 (73.4)	83 (68.0)	26 (72.2)	
Female	33 (26.6)	39 (32.0)	10 (27.8)	
Smoking	47 (37.9)	62 (50.8)	14 (38.9)	0.347
Alcohol	58 (46.8)	62 (50.8)	20 (55.6)	0.324
Family history of Gastric cancer	19 (15.3)	26 (21.3)	6 (16.7)	0.462
Previous ulcer	5 (4.0)	1 (0.8)	2 (5.6)	0.793
Histology				0.597
Low-grade dysplasia	85 (68.8)	87 (71.3)	26 (72.2)	
High-grade dysplasia	39 (31.5)	35 (28.7)	10 (27.8)	
Location				0.418
Upper 1/3	11 (8.9)	13 (10.7)	1 (2.8)	
Middle 1/3	53 (42.7)	40 (32.8)	17 (47.2)	
Lower 1/3	60 (48.4)	69 (56.6)	18 (50.0)	
Gross appearance				0.504
Elevated	117 (94.4)	106 (86.9)	20 (55.6)	
Flat	4 (3.2)	13 (10.7)	1 (2.8)	
Depressed	3 (2.4)	3 (2.5)	1 (2.8)	
Multiplicity	14 (11.3)	12 (9.8)	1 (2.8)	0.178
Maximum diameter of lesion (mm)^a^	10.84 ± 7.56	11.08 ± 8.87	10.55 ± 6.22	0.960
Metachronous recurrence^b^	19 (15.3)	10 (8.2)	7 (19.4)	0.095
Dysplasia	15 (79.0)	6 (60.0)	4 (57.2)	
Cancer	4 (21.0)	4 (40.0)	3 (42.9)	
Follow up duration (months, median, range)	53.0 (26.3–85.7)	58.3 (24.3–85.9)	57.2 (28.1–85.2)	0.026

Values are presented as mean ± SD or n (%). *H*. *pylori*, *Helicobacter pylori*.

^a^Statistical significance were tested by oneway analysis of variances among groups

^b^Statistical significance were tested by Fisher’s exact test between three groups.

**Table 2:** Comparison of clinicopathologic characteristics between patients with and without metachronous recurrence.

Characteristics	Non-metachronous group (n = 246, %)	Metachronous group (n = 36)	*P*-value
Mean age (year)	67.35 ± 8.72	70.47 ± 7.93	0.035
Sex (male)	174 (70.7)	26 (72.2)	0.854
Smoking	108 (43.9)	15 (41.7)	0.801
Alcohol	120 (48.8)	20 (55.6)	0.448
Previous ulcer	6 (2.4)	2 (5.6)	0.271
Histology			0.778
Low-grade dysplasia	172 (69.9)	26 (72.2)	
High-grade dysplasia	74 (30.1)	10 (27.8)	
Location			0.137
Upper 1/3	25 (10.2)	0 (0)	
Middle 1/3	95 (38.6)	15 (41.7)	
Lower 1/3	126 (51.2)	21 (58.3)	
Gross appearance			0.618
Elevated	223 (90.7)	34 (94.4)	
Flat	17 (6.9)	1 (2.8)	
Depressed	6 (2.4)	1 (2.8)	
Multiplicity	26 (10.6)	1 (2.8)	0.222
Maximum diameter of lesion (mm)	11.15 ± 8.08	9.17 ± 6.78	0.212

Values are presented as mean ± SD or n (%). *H*. *pylori*, *Helicobacter pylori*.

### Metachronous recurrence

**Fig 2:**
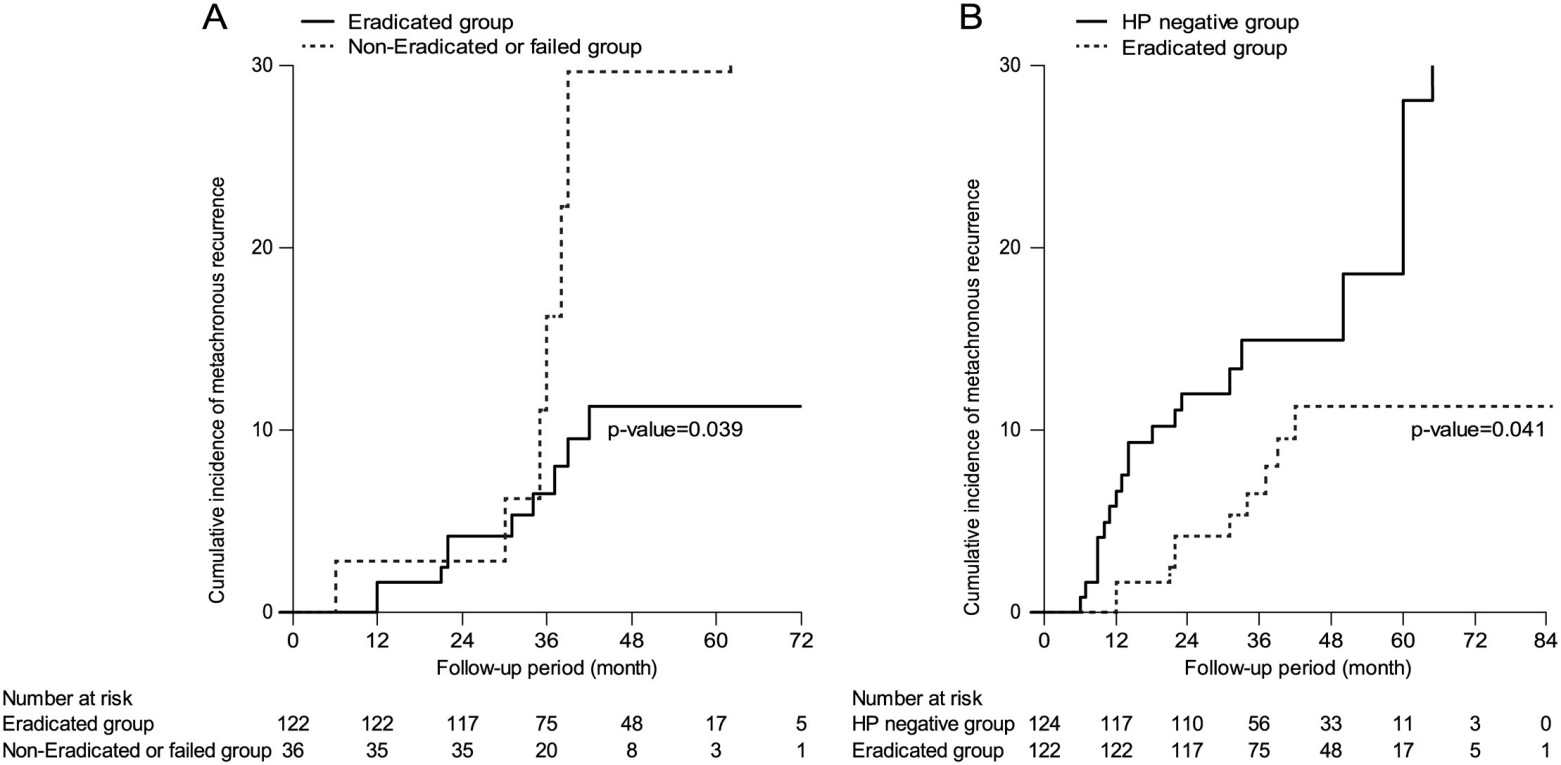
(A) The cumulative incidence of metachronous recurrence was significantly lower in the *H*. *pylori*-eradicated group in comparison with the *H*. *pylori*-persistent (non-eradicated or failed) groups (*p* = 0.039). (B) The cumulative incidence of metachronous recurrence was significantly lower in the *H*. *pylori*-eradicated group in comparison with the *H*. *pylori* negative group (*p* = 0.041).

During the follow-up period, 36 metachronous recurrences were found on endoscopy and confirmed by biopsy. Metachronous recurrence was diagnosed in 19 of 124 patients (15.3%) in the *H*. *pylori*-negative group, in 10 of 122 patients (8.2%) in the eradicated group, and in 7 of 36 patients (19.4%) in the persistent group. The median time until metachronous recurrence was 36 months (range, 6–85 months). The cumulative incidence of metachronous recurrence was significantly lower in the *H*. *pylori*-eradicated group in comparison with either of the *H*. *pylori*-persistent (non-eradicated or failed) groups (*p* = 0.039, Fig 2A). Moreover, this result was distinct by 36 months after ER. Similarly, the incidence of metachronous recurrence was significantly lower in the *H*. *pylori*-eradicated group compared with the *H*. *pylori*-negative group (*p* = 0.041, Fig 2B). Univariate analysis using Cox’s proportional hazards model showed that the persistent group had a higher risk of developing metachronous gastric neoplasms than the eradicated group (hazard ratio [HR] 3.974, *p* = 0.005).

## Discussion

According to the current Korean guidelines for treatment of *H*. *pylori* infection, eradication therapy is recommended after ER of EGC. *H*. *pylori* infection plays an important role in the development of gastric cancer. Huang *et al*. show by a meta-analysis that the odds ratio for the development of gastric cancer in *H*. *pylori*-infected patients is 1.92, and younger patients with *H*. *pylori* infection have a higher relative risk to develop gastric cancer than older patients [20]. Therefore, there is some evidence that *H*. *pylori* eradication could prevent metachronous recurrence after ER of EGC; however, data are still lacking whether there are prophylactic benefits of *H*. *pylori* eradication to prevent the development of metachronous lesions after ER of pre-malignant lesions such as gastric dysplasia.

Rapid urease test, histology, and urea breath tests are the recommended diagnostic tests for *H*. *pylori* infection. The urea breath test had high sensitivity and specificity (≥ 95%). However, false negative rates greater than 30% have been reported when antibiotics or PPI were used. For the rapid urease test, test sensitivity ranges from 85–98% and specificity ranges from 89–100%. Hematoxylin and eosin staining has a sensitivity of 69–93% and a specificity of 87–90% [17].

Recently, ER of gastric dysplasia has become accepted as a reasonable treatment option. Although the ER of low-grade dysplasia diagnosed by forceps biopsy is controversial, tumors with a size >1 cm and/or with surface redness or nodularity are believed to have significant risk for high-grade dysplasia or EGC [21]. Therefore, in Korea, ER is commonly performed for patients with both low- and high-grade dysplasia.

Since a risk of metachronous recurrence still exists in the remnant stomach following ER of gastric dysplasia, surveillance for metachronous gastric neoplasms is believed important during the follow-up period. Gutierrez-Gonzalez *et al*. show that identical genetic alterations are present in dysplasia and the surrounding intestinal metaplasia (IM) [22]. They also demonstrate that genetic changes in the IM are likely to lead to dysplasia, and further genetic alterations can cause the progression of dysplasia to gastric cancer referred to as “cancer in adenoma” [22,23]. Several causative genetic and epigenetic changes in the IM or dysplasia are reversed by *H*. *pylori* eradication. In this study, successful eradication of *H*. *pylori* infection significantly lowers metachronous recurrence; therefore, the eradication of *H*. *pylori* infection may prevent the emergence of metachronous gastric neoplasms after ER of gastric dysplasia.

There are some reports that *H*. *pylori* eradication has a prophylactic effect to prevent metachronous gastric neoplasms after ER of EGC. One large, randomized controlled trial shows a reduced incidence of metachronous gastric cancer (odds ratio [OR] 0.353, 95% confidence interval [CI] 0.161–0.775, *p* = 0.009), and recommends that prophylactic eradication should be performed after ER [12]. However, this trial doesn’t address whether there is a similar benefit to eradicate *H*. *pylori* infection after ER of gastric dysplasia. Unlike previous studies, we focused the role of *H*. *pylori* eradication in the development of metachronous lesions after ER of gastric dysplasia which was a precancerous lesion. And, in our study, *H*. *pylori* eradication could also prevent metachronous lesions after ER of gastric dysplasia, similar EGC.

*H*. *pylori* infection both initiates and promotes the gastric carcinogenesis; therefore, eradication should both inhibit newly developed gastric cancers and reduce the growth rate of existing gastric cancers [24]. After a median follow-up period of 36 months, metachronous recurrence developed in 36 patients. Although a 3-year follow-up period is short, eradication of *H*. *pylori* after ER of dysplasia might inhibit the occurrence of new gastric neoplasms and delay the growth rate of gastric cancer. Our study has some limitations. The *H*. *pylori*-negative group showed a lower metachronous recurrence rate than non-eradicated group, but a higher recurrence rate than the eradicated group. This unexpected result may be due to false negative *H*. *pylori* infection status. Additional potential causes for the false negative result might be due to antibiotic therapy administered to treat other infections, the masking effect of proton pump inhibitors (PPIs), inadequate sampling, or suboptimal staining technique [25]. We didn’t analyze the background mucosa for atrophy and intestinal metaplasia, which might also affect the rate of metachronous recurrence in the *H*. *pylori*-negative group. We didn’t consider the re-infection of *H*. *pylori*. However, five (4.1%) patients were re-infected with *H*. *pylori* in eradicated group. Therefore, the annual re-infection rate was quite low.

In conclusion, this study demonstrates that *H*. *pylori* eradication after ER in patients with gastric dysplasia may reduce the development of subsequent metachronous gastric neoplasms. Therefore, *H*. *pylori* eradication is recommended after ER of gastric dysplasia.

## References

[1] Schistosomes, liver flukes and Helicobacter pylori. IARC Working Group on the Evaluation of Carcinogenic Risks to Humans. Lyon, 7–14 June 1994. IARC Monogr Eval Carcinog Risks Hum. 1994;61: 1–241. PMC7681621 7715068

[2] Correa P. A human model of gastric carcinogenesis. Cancer Res. 1988;48: 3554–3560. 3288329

[3] de Vries AC, Haringsma J, Kuipers EJ. The detection, surveillance and treatment of premalignant gastric lesions related to Helicobacter pylori infection. Helicobacter. 2007;12: 1–15. 10.1111/j.1523-5378.2007.00475.x 17241295

[4] Schlansky B, Sonnenberg A. Epidemiology of noncardia gastric adenocarcinoma in the United States. Am J Gastroenterol. 2011;106: 1978–1985. 10.1038/ajg.2011.213 22008896

[5] Yim JY, Kim N, Choi SH, Kim YS, Cho KR, Kim SS, et al Seroprevalence of Helicobacter pylori in South Korea. Helicobacter. 2007;12: 333–340. 17669107 10.1111/j.1523-5378.2007.00504.x

[6] Woodward M, Morrison C, McColl K. An investigation into factors associated with Helicobacter pylori infection. Journal of Clinical Epidemiology. 2000;53: 175–181. 10729690 10.1016/s0895-4356(99)00171-7

[7] Everhart JE, Kruszon-Moran D, Perez-Perez GI, Tralka TS, McQuillan G. Seroprevalence and ethnic differences in Helicobacter pylori infection among adults in the United States. Journal of Infectious Diseases. 2000;181: 1359–1363. 10762567 10.1086/315384

[8] Kim YJ, Park JC, Kim JH, Shin SK, Lee SK, Lee YC, et al Histologic diagnosis based on forceps biopsy is not adequate for determining endoscopic treatment of gastric adenomatous lesions. Endoscopy. 2010;42: 620–626. 10.1055/s-0030-1255524 20623445

[9] Kang KJ, Lee JH. Characteristics of Gastric Cancer in Korea—with an Emphasis on the Increase of the Early Gastric Cancer (EGC). Journal of the Korean Medical Association. 2010;53: 283–289.

[10] Nasu J, Doi T, Endo H, Nishina T, Hirasaki S, Hyodo I. Characteristics of metachronous multiple early gastric cancers after endoscopic mucosal resection. Endoscopy. 2005;37: 990–993. 16189772 10.1055/s-2005-870198

[11] Isomoto H, Shikuwa S, Yamaguchi N, Fukuda E, Ikeda K, Nishiyama H, et al Endoscopic submucosal dissection for early gastric cancer: a large-scale feasibility study. Gut. 2009;58: 331–336. 10.1136/gut.2008.165381 19001058

[12] Fukase K, Kato M, Kikuchi S, Inoue K, Uemura N, Okamoto S, et al Effect of eradication of Helicobacter pylori on incidence of metachronous gastric carcinoma after endoscopic resection of early gastric cancer: an open-label, randomised controlled trial. Lancet. 2008;372: 392–397. 10.1016/S0140-6736(08)61159-9 18675689

[13] Bae SE, Jung HY, Kang J, Park YS, Baek S, Jung JH, et al Effect of Helicobacter pylori eradication on metachronous recurrence after endoscopic resection of gastric neoplasm. Am J Gastroenterol. 2014;109: 60–67. 10.1038/ajg.2013.404 24343545

[14] Kim YI, Choi IJ, Kook MC, Cho SJ, Lee JY, Kim CG, et al The association between Helicobacter pylori status and incidence of metachronous gastric cancer after endoscopic resection of early gastric cancer. Helicobacter. 2014;19: 194–201. 10.1111/hel.12116 24612125

[15] Maehata Y, Nakamura S, Fujisawa K, Esaki M, Moriyama T, Asano K, et al Long-term effect of Helicobacter pylori eradication on the development of metachronous gastric cancer after endoscopic resection of early gastric cancer. Gastrointest Endosc. 2012;75: 39–46. 10.1016/j.gie.2011.08.030 22018552

[16] Choi J, Kim SG, Yoon H, Im JP, Kim JS, Kim WH, et al Eradication of Helicobacter pylori after endoscopic resection of gastric tumors does not reduce incidence of metachronous gastric carcinoma. Clin Gastroenterol Hepatol. 2014;12: 793–800.e791 10.1016/j.cgh.2013.09.057 24100112

[17] Kim SG, Jung HK, Lee HL, Jang JY, Lee H, Kim CG, et al Guidelines for the diagnosis and treatment of Helicobacter pylori infection in Korea, 2013 revised edition. J Gastroenterol Hepatol. 2014 2014/04/25. 10.1111/jgh.12607 24758240

[18] Japanese classification of gastric carcinoma: 3rd English edition. Gastric Cancer. 2011;14: 101–112. 10.1007/s10120-011-0041-5 21573743

[19] Stolte M. The new Vienna classification of epithelial neoplasia of the gastrointestinal tract: advantages and disadvantages. Virchows Arch. 2003;442: 99–106. 12596058 10.1007/s00428-002-0680-3

[20] Huang JQ, Sridhar S, Chen Y, Hunt RH. Meta-analysis of the relationship between Helicobacter pylori seropositivity and gastric cancer. Gastroenterology. 1998;114: 1169–1179. 9609753 10.1016/s0016-5085(98)70422-6

[21] Choi CW, Kim HW, Shin DH, Kang DH, Hong YM, Park JH, et al The risk factors for discrepancy after endoscopic submucosal dissection of gastric category 3 lesion (low grade dysplasia). Dig Dis Sci. 2014;59: 421–427. 10.1007/s10620-013-2874-8 24366779

[22] Gutierrez-Gonzalez L, Graham TA, Rodriguez-Justo M, Leedham SJ, Novelli MR, Gay LJ, et al The clonal origins of dysplasia from intestinal metaplasia in the human stomach. Gastroenterology. 2011;140: 1251–1260 e1251–1256. 10.1053/j.gastro.2010.12.051 21223968

[23] Sugano K. Premalignant conditions of gastric cancer. Journal of Gastroenterology and Hepatology. 2013;28: 906–911. 10.1111/jgh.12209 23560829

[24] Kato M, Asaka M, Ono S, Nakagawa M, Nakagawa S, Shimizu Y, et al Eradication of Helicobacter pylori for primary gastric cancer and secondary gastric cancer after endoscopic mucosal resection. Journal of Gastroenterology. 2007;42: 16–20. 10.1007/s00535-006-1928-5 17238020

[25] Genta RM, Lash RH. Helicobacter pylori-negative gastritis: seek, yet ye shall not always find. Am J Surg Pathol. 2010;34: e25–34. 10.1097/PAS.0b013e3181e51067 20631607

